# Ethnic background and children’s oral health-related quality of life

**DOI:** 10.1007/s11136-019-02159-z

**Published:** 2019-03-11

**Authors:** A. W. van Meijeren-van Lunteren, E. B. Wolvius, H. Raat, V. W. V. Jaddoe, L. Kragt

**Affiliations:** 1 Department of Oral & Maxillofacial Surgery, Special Dental Care and Orthodontics, Erasmus University Medical Center, P.O Box 2040, 3000 CA Rotterdam, The Netherlands; 2 The Generation R Study Group, Erasmus University Medical Center, Rotterdam, The Netherlands; 3 Department of Public Health, Erasmus University Medical Center, Rotterdam, The Netherlands; 4 Department of Pediatrics, Erasmus University Medical Center, Rotterdam, The Netherlands; 5 Department of Epidemiology, Erasmus University Medical Center, Rotterdam, The Netherlands

**Keywords:** Quality of life, Dental health perception, Ethnicity, Disparities, Public health

## Abstract

**Purpose:**

Ethnic background is known to be related to oral health and socioeconomic position (SEP). In the context of patient-centered oral health care, and the growing number of migrant children, it is important to understand the influence of ethnic background on oral health-related quality of life (OHRQoL). Therefore, we aimed to identify the differences in children’s OHRQoL between ethnic groups, and the contribution of oral health status, SEP, and immigration characteristics.

**Methods:**

This study was part of the Generation R Study, a prospective cohort study conducted in Rotterdam, the Netherlands. In total, 3121 9-year-old children with a native Dutch (*n* = 2510), Indonesian (*n* = 143), Moroccan (*n* = 104), Surinamese (*n* = 195), or Turkish (*n* = 169) background participated in the present study. These ethnicities comprise the most common ethnic groups in the Netherlands. OHRQoL was assessed using a validated short form of the child oral health impact profile. Several regression models were used to study an association between ethnic background and OHRQoL, and to identify potential mediating factors.

**Results:**

Turkish and Surinamese ethnic background were significantly associated with lower OHRQoL. After adjusting for mediating factors, only Surinamese children had a significantly lower OHRQoL than Dutch children (β:− 0.61; 95% CI− 1.18 to –0.04).

**Conclusions:**

Our results show that Turkish and Surinamese children have a significantly lower OHRQoL than native Dutch children. The association was partly explained by oral health status and SEP, and future studies are needed to understand (cultural) the determinants of ethnic disparities in OHRQoL, in order to develop effective oral health programs targeting children of different ethnic groups.

**Electronic supplementary material:**

The online version of this article (10.1007/s11136-019-02159-z) contains supplementary material, which is available to authorized users.

## Introduction

Oral health status in children has been improved over the past few decades. However, differences in the prevalence of several clinical oral health outcomes still exist in western countries, including the Netherlands, disfavoring children from ethnic minority groups [1–3]. At present, there is no full understanding of the pathway driving these oral health inequalities [4]. In the last 20 years, the proportion of migrants in the Dutch population increased from 17 to 23% [5]. Considering the internationalization and the increase of migration groups, the number of children with a non-native background will grow further in the future, and the possibility of oral health inequalities might increase [6].

Oral health cannot be disclosed from the broader framework of general health. The mouth allows us to speak, smile, and eat every day. Any impairment in its functioning will limit people in social-psychological circumstances, and also has a proven relationship with individuals’ systemic health system [7]. Therefore, social, psychological, and functional factors should be considered in relation to oral health [8, 9]. This notion originates since the late twentieth century when a shift has occurred from the clinical towards patient perspective regarding oral health, and the increasingly more important role of patient participation in oral health treatments. Several patient-reported outcome measures have been evolved since that time, including oral health-related quality of life (OHRQoL) [10, 11]. OHRQoL is a concept based on the individual and subjective perception of oral health and oral diseases, and measures its impact in individuals. OHRQoL can give more understanding of the self-perceived oral health, oral health behaviors, and health care needs, which in turn may influence individual oral health status [9, 12]. Therefore, it is important to measure and identify OHRQoL in children, especially considering that poor oral health during childhood will continue during adulthood [13].

Ethnic background may be one of the factors influencing OHRQoL in children, partly mediated through oral health status. It is known that ethnic background is strongly related to socioeconomic position (SEP), which has been shown to indirectly influence children’s OHRQoL [2, 14–17]. In addition, cultural background of children and their families might influence how children perceive their oral health [18–20]. In this context, immigration characteristics, such as language proficiency and age of the mother at immigration, might be possible indicators of the existing influence of the cultural background on health [21].

Therefore, the aim of this research was to identify differences in OHRQoL between ethnic groups, in a population of children born in Rotterdam, the Netherlands. Additionally, we assessed whether potential differences in OHRQoL can be explained by oral health status, socioeconomic factors, and immigration characteristics.

## Methods

### Study population

This study was part of the Generation R study, a population-based multi-ethnic cohort study from fetal life onwards, conducted in Rotterdam, the Netherlands. The Generation R Study has been described in detail elsewhere [22]. This study was conducted in accordance with the guidelines proposed in the World Medical Association‘s Declaration of Helsinki and has been approved by the Medical Ethical Committee at the Erasmus University Medical Center, Rotterdam, the Netherlands (MEC-2012-165). All participants gave written informed consent before any measurement started.

Invitations to participate in the Generation R study were made to all pregnant women in the study area between April 2002 and January 2006. In total, 9749 children participated in the first phase of the study. The present study is conducted in the follow-up phase when the children were 9 years old and in which 7393 children of the cohort participated. For the purpose of the present study, we excluded children without information on OHRQoL due to no or a limited response to questionnaire (*n* = 3597). Furthermore, we only included children representing the four major ethnic groups (*n* > 100) of the study population, remaining with an final study population of *n* = 3121.

### Ethnic background and immigration characteristics

All children were born in Rotterdam, the Netherlands, and for ethnic background we took into account maternal ethnicity, since mothers are most often primary caretakers. We made use of the Dutch classification of ethnic background, which was determined by mothers’ parents places of birth [23]. If both parents of the mother were born in the Netherlands, ethnic background was classified as Dutch. If one of the parents was born in another country than the Netherlands, ethnic background was classified as non-Dutch. If both parents were born in a different country, maternal country of birth defined the ethnic background. Information about mothers’ parents places of birth was retrieved via questionnaires at enrolment in the Generation R study and when the child was 5 years old.

Characteristics about immigration of the mother were retrieved via questionnaires at enrolment in the Generation R study. Mothers with another ethnic background than Dutch were further classified as first-generation and second-generation mothers. First-generation mothers were then again subdivided in two categories based on age of immigration (0–15 years and ≥ 16 years). We assume that mothers in the latter group did not attend school in the Netherlands, since that is not mandatory for children within that age category. We also assessed language proficiency of the mothers within three questions about speaking, reading, and writing skills. The three 5-point scales answers were summed up and categorized into: worse (3–9 points), reasonable (10–14 points), and good (15 points) Dutch language skills.

### OHRQoL

Children’s OHRQoL was assessed using a validated short form of the Child Oral Health Impact Profile (COHIP-ortho or COHIP-11). The COHIP-ortho is a derivation of the COHIP-38, and aimed to easily and shortly assess OHRQoL in children, especially in large-scale epidemiologic studies [24]. Parents filled in the COHIP instrument when the children were 9 years old. This short form COHIP measure is a questionnaire that measures OHRQoL of the child with 11 questions, covering the different domains of oral health, including oral health status, social-emotional wellbeing, functional wellbeing, school and peer interaction, and self-image (Online Resource Table 1). Questions were answered on five-point Likert scale items (1: Almost all the time; 2: Fairly often; 3: Sometimes; 4: Almost never; 5: Never), and the scores per question were finally summed up for every individual. The total overall score ranged from 11 to 55, with higher scores corresponding to higher reported OHRQoL. If there were less than three missing values in the responses to the OHRQoL questionnaire, the missing values were replaced by the personal mean score of the remaining answers to the questions, as it is proposed by other researchers that used the original version of this questionnaire [25].

### Confounders and mediators

Child’s gender and age were considered factors that potentially confound the association between ethnic background and OHRQoL. Information about these variables was assessed via questionnaires. Oral health status (caries experience) and SEP were considered as potential meditating factors in the pathway from ethnic background to OHRQoL.

Dental caries experience was assessed with the decayed, missing, and filled teeth index (dmft) [26]. The dmft index of each participating child was obtained with intraoral photographs captured by trained research personnel and dental students during a visit at the research center of Generation R when children were around 5 years old [1, 27]. Generally, the dmft index ranges from 0 to 20, and for statistical analyses; the dmft index was categorized into three groups. The three categories were based on the mean dmft index of 5-year-old Dutch children (mean ± SD: 1.6 ± 2.5): no caries experience (dmft = 0), mild caries experience (dmft = 1–3), and severe caries experience (dmft ≥ 3) [28].

SEP was assessed with two common indicators: maternal education level and net household income. Information about these indicators was derived from questionnaires during enrollment in the study, and when children were around 5 years old. Educational level was defined into three categories according to Statistics Netherlands : low (no education, primary education, or secondary phase 1 finished), middle (secondary phase 2 finished), and high (higher vocational training or a university degree finished) [29]. Net household income was categorized into three groups, based on the division of total spendable income in 2014 of Dutch households: < 1600; 1600–3600, > 3600 [30].

### Statistical analyses

Descriptive statistics were calculated per ethnic group to characterize the study population, and reported as follows: means with standard deviation (SD) for continuous variables, and absolute numbers (*n*) with percentages (%) for categorical variables. To test whether the individual and family characteristics differed significantly between the five ethnic groups, *p* values were derived using One-Way ANOVA and Chi-Square tests. Based on the mean OHRQoL per ethnic background group, Cohen’s effect sizes (d) were used to get an impression of the important differences in OHRQoL in our study. Cohen’s effect sizes were calculated by dividing the difference in mean OHRQoL scores among ethnic groups by a pooled SD, and accordingly interpreted as follows: 0.2 ≤ d < 0.5 small difference, 0.5 ≤ d < 0.8 moderate difference, d ≥ 0.8 large difference [31] (Online Resource Table 2).

To study the associations between ethnic background and OHRQoL, weighted linear regression models were used to estimate the change in the total overall OHRQoL score, in Indonesian, Moroccan, Surinamese, and Turkish children compared to Dutch children. We used weighted linear regression after evaluating the assumptions of linear regression analyses, and observing heteroscedasticity in our data. Besides the unadjusted model, multivariable analyses were used. The first model included the following potential confounding variables: gender and age of the child. To determine the following models, we prior evaluated mediating effects (M) by using the Baron and Kenny method [32].

The association between ethnic background (X) and OHRQoL (Y) was assessed using the results of model 1 between ethnic background and OHRQoL provided in Table 2.

1. The association between ethnic background (X) and oral health status (M) and SEP (M) was assessed using the results of the descriptive statistics.
2. The association between oral health status (M) and SEP (M) and OHRQoL (Y) was assessed using several weighted linear regression models, determining the relation between oral health status and OHRQoL, and between SEP and OHRQoL, adjusted for age and gender (Online Resource Table 3).
3. When all assumptions above held, the potential mediators were added to the models, separately and combined. Changes in percentages were calculated for the adjustment of each factor by ([β_adjusted model_−β_model 1_]/[β_model 1_]*100).

Also, analyses were performed in subgroups of children with a Surinamese background, and effect estimates were calculated for Surinamese Creole and Surinamese Hindustani children.

To assess the relation between immigration characteristics and OHRQoL, univariate weighted linear regression models were used to estimate the change in OHRQoL per immigration characteristic, stratified for ethnic background groups. Results based on a subgroup sample size of *n* < 10 were not interpreted due to low statistical power.

Furthermore, ordinal logistic regression models were used to estimate the Odds Ratios (OR) for each of the 11 Likert scale item separately, to estimate the difference per item of the questionnaire in Indonesian, Moroccan, Surinamese, and Turkish children compared to Dutch children (Online Resource Table 4).

Individual and family characteristics of children with missing data on OHRQoL and other ethnic backgrounds (excluded, *n* = 4272) were compared with children participating in the current study (included, *n* = 3121). This non-response analysis indicated that the children included in the present study tend to have less caries experience and higher SEP indicated by maternal educational level and family household income (Online Resource Table 5).

Because of the missing data in the covariates, the Markov Chain Monte Carlo (MCMC) method was applied to generate a dataset based on ten imputations. Imputations were based on all variables in the models, but the main determinant, (ethnic background) and outcome (children’s OHRQoL) were not imputed. We presented the pooled estimates of these datasets in the results. Statistical analyses were conducted using SPSS (IBM Corp. Released 2016. IBM SPSS Statistics for Windows, Version 24.0. Armonk, NY: IBM Corp.).

Sensitivity analyses were performed in datasets based on 15 and 20 imputations. Results were compared, and similar results were observed (data not shown). Also, OHRQoL of children with a different background than their mothers was compared with the OHRQoL of children having the same ethnic background as their mothers. Additionally, a comparison was made between OHRQoL of children whose fathers had a different ethnic background than mothers (Online Resource Table 6). A two-tailed *p *value of *p* < 0.05 was considered significant for all analyses.

## Results

The study population consisted of 3121 children: 2510 native Dutch, 143 Indonesian, 104 Moroccan, 195 Surinamese, and 169 Turkish children. Surinamese children were categorized in Creole (*n* = 72) and Hindustani (*n* = 84) (Online Resource Fig. 1).

The prevalence of a caries-free dentition was higher among native Dutch children (80.2%) and Indonesian children (83.2%) than among Moroccan (39.0%), Surinamese (68.3%), and Turkish children (49.2%). OHRQoL of native Dutch (mean 49.2; SD 3.0) and Indonesian children (mean 49.1; SD 2.9) was higher than OHRQoL of Moroccan (mean 48.7; SD 3.6), Surinamese (mean 48.4; SD 3.7), and Turkish children (mean 48.2; SD 4.1). Generally, Dutch children had a higher SEP as indicated by maternal education and household income (Table 1).

**Table 1:** Characteristics of the total study population presented by ethnic background (*N* = 3121)

	Dutch	Indonesian	Moroccan	Surinamese	Turkish	*p* value
	*N* = 2510	*N* = 143	*N* = 104	*N* = 195	*N* = 169	
Individual characteristics						
Child’s gender						0.947
Boys, *n* (%)	1262 (50.3)	69 (48.3)	55 (52.9)	99 (50.8)	88 (52.1)	
Girls, *n* (%)	1248 (49.7)	74 (51.7)	49 (47.1)	96 (49.2)	81 (47.9)	
Child’s age						< 0.001
Mean (SD)	9.8 (0.3)	9.8 (0.4)	9.9 (0.4)	9.9 (0.4)	10.0 (0.5)	
Child’s caries experience, *n* (%)						< 0.001
No caries (dmft 0)	1496 (80.2)	89 (83.2)	32 (39.0)	97 (68.3)	64 (49.2)	
Mild caries (dmft 1–3)	278 (14.9)	14 (13.1)	23 (28.0)	32 (22.5)	21 (16.2)	
Severe caries (dmft > 3)	92 (4.9)	4 (3.7)	27 (32.9)	13 (9.2)	45 (34.6)	
Missings,* n* (%)	*644 (25.7)*	*36 (25.2)*	*22 (21.2)*	*53 (27.2)*	*39 (23.1)*	
Child’s OHRQoL						< 0.001
Mean (SD)	49.2 (3.0)	49.1 (2.9)	48.7 (3.6)	48.4 (3.7)	48.2 (4.1)	
Family characteristics						
Maternal education level, *n* (%)						< 0.001
Low	220 (9.0)	7 (5.0)	30 (34.9)	44 (24.3)	65 (42.5)	
Middle	627 (25.6)	36 (25.9)	35 (40.7)	91 (50.3)	63 (41.2)	
High	1607 (65.5)	96 (69.1)	21 (24.4)	46 (25.4)	25 (16.3)	
Missings,* n* (%)	*56 (2.2)*	*4 (2.8)*	*18 (17.3)*	*14 (7.2)*	*16 (9.5)*	
Household income, *n* (%)						< 0.001
Low (< €1600)	156 (6.6)	10 (7.5)	41 (44.1)	49 (27.5)	52 (34.4)	
Middle (€1600–3600)	775 (32.8)	53 (39.6)	42 (45.2)	82 (46.1)	80 (53.0)	
High (> €3600)	1432 (60.6)	71 (53.0)	10 (10.8)	47 (26.4)	19 (12.6)	
Missings,* n* (%)	*147 (5.9)*	*9 (6.3)*	*11 (10.6)*	*17 (8.7)*	*18 (10.7)*	
Generational status, *n* (%)						< 0.001
First generation		13 (9.3)	77 (82.8)	136 (74.7)	113 (71.1)	
Second generation		127 (90.7)	16 (17.2)	46 (25.3)	46 (28.9)	
Missings, *n* (%)		*3 (2.1)*	*11 (10.6)*	*13 (6.7)*	*10 (5.9)*	
Age of immigration, *n* (%)						0.145
< 15 year		6 (46.2)	33 (49.3)	74 (60.2)	42 (45.2)	
> 15 year		7 (53.8)	34 (50.7)	49 (39.8)	51 (54.8)	
Missings, n (%)		*130 (90.9)*	*37 (35.6)*	*72 (36.9)*	*76 (45.0)*	
Language skills, *n* (%)						< 0.001
Worse		3 (2.4)	22 (28.9)	1 (0.6)	42 (31.6)	
Reasonable		8 (6.4)	22 (28.9)	36 (22.1)	52 (39.1)	
Good		114 (91.2)	32 (42.1)	126 (77.3)	39 (29.3)	
*Missings, n (%)*		*18 (12.6)*	*28 (26.9)*	*32 (16.4)*	*36 (21.3)*	

Italic printed effect estimates have *n* < 10

Numbers are presented as absolute numbers for categorical variables or as mean (SD) for continuous variables. P-values are estimated based on one-way ANOVAs and Chi-square tests

Surinamese and Turkish children had significantly lower OHRQoL than native Dutch children after adjustments for confounding variables (Model 1: Surinamese: β:− 0.75; 95% CI − 1.32 to − 0.18; Turkish: β:− 1.00; 95% CI − 1.64 to − 0.35). This difference remained significant after adjustments for oral health status (Model 2: Surinamese: β:− 0.70; 95% CI − 1.27 to − 0.13; Turkish: β:− 0.74; 95% CI − 1.40 to  − 0.09) or SEP (Model 3: Surinamese: β:− 0.63; 95% CI − 1.20 to − 0.05; Turkish: β:− 0.74; 95% CI − 1.40 to − 0.08). After adjustments for both mediators (oral health status and SEP), a statistically significant association between ethnic background and OHRQoL solely remained for Surinamese children (Model 4: β:− 0.61; 95% CI − 1.18 to − 0.04). The mediating effects of oral health status and SEP combined for Moroccan, Surinamese, and Turkish children were, respectively, − 81.3%, − 18.9%, and − 42.0% (Table 2).

**Table 2:** Weighted linear regression models showing the associations between ethnic background (Indonesian, Moroccan, Surinamese, Turkish, and Dutch), and OHRQoL

	Model 1	Model 2	Change i (%)	Model 3	Change ii (%)	Model 4	Change iii (%)
Dutch (*n* = 2510)	Ref	Ref		Ref		Ref	
Indonesian (*n* = 143)	− 0.04 (− 0.54 to 0.47)	− 0.07 (− 0.57 to 0.43)	+ 75.0	− 0.04 (− 0.54 to 0.46)	0.0	− 0.07 (− 0.57 to 0.44)	+ 75.0
Moroccan (*n* = 104)	− 0.48 (− 1.18 to 0.23)	− 0.18 (− 0.90 to 0.54)	− 62.5	− 0.30 (− 1.02 to 0.42)	− 37.5	− 0.09 (− 0.82 to 0.64)	− 81.3
Surinamese (*n* = 195)	− **0.75 (**− **1.32 to − 0.18)**^a^	− **0.70 (**− **1.27 to − 0.13)**^a^	− 6.7	− **0.63 (**− **1.20 to − 0.05)**^a^	− 16.0	− **0.61 (**− **1.18 to − 0.04)**^a^	− 18.9
Turkish (*n* = 169)	− **1.00 (**− **1.64 to − 0.35)**^b^	− **0.74 (**− **1.40 to − 0.09)**^a^	− 26.0	− **0.74 (**− **1.40 to − 0.08)**^a^	− 26.0	− 0.58 (− 1.24 to 0.09)	− 42.0

Bold printed effect estimates are statistically significant

Data are presented as weighted least squares regression coefficients (β) with 95% confidence interval (95% CI)

Model 1 is adjusted for age and gender of the child, Model 2 is additionally adjusted for caries experience, Model 3 is additionally adjusted for family income and educational level of the mother, Model 4 is additionally adjusted for caries experience, family income, and educational level of the mother. Change I, Change ii, and Change iii display the change in effect estimate after adjustment for mediating factors relative to Model 1: ([β adjusted model - β model 1]/[β model 1]*100)

^a^*p* < 0.05

^b^*p* < 0.01

After stratifying Surinamese children into Hindustani and Creole (Table 3), the association between OHRQoL and ethnic background was solely significant for Surinamese Hindustani children (Model 3: β:− 1.19; 95% CI− 2.15 to − 0.23). In addition, Table 4 shows that children of second-generation mothers with a Surinamese background have a higher OHRQoL than children of first-generation mothers with a Surinamese background (β:1.37; 95% CI 0.40 to 2.33).

**Table 3:** Weighted linear regression models showing the associations between ethnic background (Surinamese Creole, Surinamese Hindustani, and Dutch) and OHRQoL

	Model 1	Model 2	Model 3
Dutch (*n* = 2510)	Ref	Ref	Ref
Surinamese Creole (*n* = 72)	0.02 (− 0.76 to 0.80)	0.06 (− 0.78 to 0.79)	0.24 (− 0.53 to 1.01)
Surinamese Hindustani (*n* = 84)	− **1.35 (**− **2.31 to − 0.39)**^b^	− **1.25 (**− **2.20 to − 0.30)**^a^	− **1.19 (**− **2.15 to − 0.23)**^a^

Bold printed effect estimates are statistically significant

Data are presented as weighted least squares regression coefficients (β) with 95% confidence interval (95% CI)

Model 1 is adjusted for age and gender of the child, Model 2 is additionally adjusted for caries experience, Model 3 is additionally adjusted for caries experience, family income, and educational level of the mother

^a^*p* < 0.05

^b^*p* < 0.01

**Table 4:** Weighted linear regression models showing the associations between maternal immigration characteristics and OHRQoL, stratified by ethnic background

	Indonesian	Moroccan	Surinamese	Turkish
Generational status (*n* = 574)				
First generation (*n* = 339)	Ref	Ref	Ref	Ref
Second generation (*n* = 235)	1.37 (− 0.43 to 3.16)	− **2.03 (**− **3.65 to − 0.42)**^a^	**1.37 (0.40 to –2.33)** ^a^	− 0.53 (− 2.03 to 0.96)
Age at immigration ( ***n*** = **296)**				
0–15 (*n* = 155)	Ref	Ref	Ref	Ref
≥ 16 (*n* = 141)	− *1.21 (*− *4.58 to 2.15)*	0.09 (− 1.52 to 1.70)	− 0.05 (− 1.52 to 1.42)	0.18 (− 1.30 to 1.65)
Dutch language skills ( ***n*** = **497)**				
Good (*n* = 311)	Ref	Ref	Ref	Ref
Reasonable (*n* = 118)	− *0.93 (*− *2.57 to 0.72)*	− 0.55 (− 2.37 to 1.28)	− 0.70 (− 2.12 to 0.71)	− 0.70 (− 2.33 to 0.93)
Worse (*n* = 68)	− *3.84 (*− *9.34 to 1.66)*	− 0.55 (− 2.45 to 1.36)	− ***6.84 (***− ***7.44 to − 6.25)***^a^	− 0.07 (− 1.74 to 1.60)

Bold printed effect estimates are statistically significant and italic printed effect estimates indicate a sample size of *n* < 10

Data are presented as weighted least squares regression coefficients (β) with 95% confidence interval (95% CI)

^a^*p* < 0.05

## Discussion

The results of this study, among a multi-ethnic population in Rotterdam, show that OHRQoL is perceived lower in children with a Turkish and Surinamese ethnic background than in native Dutch children; in Surinamese children, this was independent of oral health status and SEP.

A part of the observed association between Surinamese ethnic background and OHRQoL was mediated by oral health status and SEP. In Moroccan and Turkish children, the mediating effects of oral health status and SEP were higher, and explained the association in Turkish children. On its turn, this can be explained by higher caries experience and lower SEP in Moroccan and Turkish children. Also from literature, it is known that oral health status and socioeconomic indicators influence OHRQoL [16, 17]. Sill, more underlying mechanisms need to be discussed to explain the observed association in children with a Surinamese ethnic background.

In general, cultural norms and level of integration in the Dutch society influence the perception of health, and could therefore lead to a different OHRQoL among children from ethnic minority groups [33]. Where children with Indonesian mothers, who are mostly second generation, have no lower OHRQoL, we demonstrated that Surinamese mothers are mostly first-generation immigrants, and have a lower OHRQoL. Stratified analyses show that solely Surinamese Hindustani children have a significantly lower OHRQoL. Surinamese Hindustani immigrants face more difficulties with integrating into the new Dutch culture, and accepting cultural norms, which might explain the results we found [34]. However, we should be aware that ethnic background is a complex concept, which involves elements of culture, religion, and migration history; therefore, other cultural and social factors, which were not assessed in the present study, might underlie the relation between ethnic background and OHRQoL as well.

So far, limited research is performed in the specific area of ethnic background and children’s OHRQoL. A study in Brazil has shown that non-white children had a significantly lower OHRQoL compared to white children [35]. In this study, the differences in OHRQoL were mainly based on emotional and social wellbeing, whereas in our study mainly the oral symptoms were responsible for the difference (Online Resource Table 4). Although we are comparing two different societies and dissimilar ethnic circumstances, still both studies show a lower perceived OHRQoL in children of ethnic minority groups. Differences in OHRQoL were also found between Hispanic and white elderly in the US [36]. Recently, differences in OHRQoL were studied between groups with and without a migration background, in a German population which was based on clinical orthodontic patients and students [37]. In accordance with our study, they have shown that children and adolescents with a migration background score lower OHRQoL than non-migrants, which was partly explained by socioeconomic and oral health status.

The results of our study have to be seen in the light of some limitations. For our study, we used the instrument COHIP-ortho, this questionnaire was initially developed to assess the OHRQoL in children with orthodontic treatment(-need). However, the original COHIP-38 was validated in a diverse sample of children representing a variety of oral conditions and ethnicities [38]. Also, we assume that children with orthodontic treatment(-need) are comparable with children without orthodontic treatment(-need), with respect to filling in the COHIP-ortho questionnaire, which justifies the use of the COHIP-ortho in this study. Another critical point is that parents answered the COHIP-ortho. Parents might have limited knowledge on social and emotional characteristics of children, and although several studies verified parents as useful and reliable proxies to study children’s OHRQoL, the effect estimates might slightly be diluted due to random misclassification of children’s OHRQoL [33, 39, 40]. Lastly, selection bias could have played a role in the current study when observing the non-response rates and characteristics of non-participants. Non-participants tend to have more caries experience, and lower SEP indicated by maternal education level, which could have led to a underestimation of our results (Online Resource Table 5). Also, dental caries, used as covariate in the analyses, was assessed using intraoral photographs. This method is not optimal for assessing dental caries in a clinical setting, but it seemed to be suitable for scientific studies [27]. Still, the results of our study might be affected by a non-differential misclassification due to an underestimation of dental caries in our study population, which might have caused a slightly overestimation of the effect estimates. Furthermore, the effect estimates in our study, showing the absolute decrease in OHRQoL, are rather small. Unfortunately, no information exists about the minimal important difference (MID) of the COHIP instrument we used to assess OHRQoL [41]. However, when interpreting the Cohen’s effect sizes (Online Resource Table 2) small differences were found [31]. This is partly caused by a ‘ceiling effect’: a high percentage of the study population reported positive OHRQoL scores. This phenomenon is common in quality-of-life research and limits to observe MIDs in a generally healthy study population [42]. However, taking into account the use of OHRQoL, the results of this study might still be interesting for clinical practice. For instance, it indicates that the quality of life of some ethnic minority groups is not defined by simply their health status [9, 11].

The major strength of this study is the use of such a large multi-ethnic population-based cohort. To the best of our knowledge, this was the first population-based study investigating the relationship between ethnic background of children in relation to OHRQoL. Concerning ethnic background, it is important to insure validity of the ethnic classification. Since no gold standard for ethnic background exists, this often stresses difficulties, and can lead to incorrect results. In the current study, we were able to classify ethnic background in a proper systematic way, proposed by Statistics Netherlands [23]. We made use of the ethnic background of the mother, considered as the main caregiver of a child. This concept of ‘country of birth’ is objective, stable, and it allows for comparability between groups. In addition, previous studies have shown that the concept ‘country of birth’ is highly related to ‘self-identified’ ethnic background [43]. However, using country of birth does not necessarily address the multifaceted aspects of ethnicity. People born in the same country might still have differences in language, religion, or culture [44]. However, we have tried to capture this issue by providing other immigration characteristics, per ethnic group and in relation to OHRQoL, which gives more insight into the mechanism behind ethnic background of mothers and OHRQoL of children. Results would not have been different taking into account the ethnic background of children, or fathers (Online Resource Table 6); however, no Indonesian children would have been included then. In addition, using maternal ethnic background supported the use of SEP of mothers and families as mediators of the association. As part of such a large cohort study, we were able to adjust our regression models for several well-measured covariates in order to minimize the risk of confounding bias. We assume that any other information bias that could have occurred during the study, e.g., recall bias or asymmetric measurements, is on a random basis and could only led to a dilution of the associations observed.

## Conclusion

The current population-based study shows that ethnic differences in OHRQoL exists, and that having a Surinamese and Turkish ethnic background negatively influences OHRQoL. Main explanatory factors for low OHRQoL in children of ethnic minorities are oral health status and SEP, but in Surinamese children other unknown factors are related to low OHRQoL. Low OHRQoL potentially influences oral health behavior leading to future oral health impairment. Especially, when considering the increasing number of migration groups in the Netherlands, it is important to understand and identify further (cultural) factors related to oral health and OHRQoL. Therefore, in-depth studies focusing on potential (cultural) determinants of ethnic disparities in children’s oral health are highly recommended. Accordingly, effective preventive strategies can be developed to target children of different ethnic and cultural groups, and to raise awareness among care providers and policy makers.

## Electronic supplementary material

Below is the link to the electronic supplementary material.

Supplementary material 1 (DOCX 63 KB)
