# Supplementary material for: Ethnic background and children’s oral health-related quality of life
Source: Qual Life Res. 2019 Mar 11;28(7):1783–91. doi: 10.1007/s11136-019-02159-z (PMC6571084; doi:10.1007/s11136-019-02159-z)
Supplement: Supplementary file 1 — Supplementary material 1 (DOCX 63 KB) [file 11136_2019_2159_MOESM1_ESM.docx]

# Electronic Supplementary Material

***Ethnic Background and Children’s Oral Health Related Quality of Life***

**A.W. van Meijeren - van Lunteren, MSc**; Department of Oral & Maxillofacial Surgery, Special Dental Care and Orthodontics, Erasmus University Medical Center, Rotterdam, the Netherlands; The Generation R Study Group, Erasmus University Medical Center, Rotterdam, the Netherlands; a.vanlunteren@erasmusmc.nl

**E. B. Wolvius, DDS, MD, PhD, Prof**; Department of Oral & Maxillofacial Surgery, Special Dental Care and Orthodontics, Erasmus University Medical Center, Rotterdam, the Netherlands; The Generation R Study Group, Erasmus University Medical Center, Rotterdam, the Netherlands

**H. Raat, MD, PhD, MBA, Prof**; Department of Public Health, Erasmus University Medical Center, Rotterdam, the Netherlands; The Generation R Study Group, Erasmus University Medical Center, Rotterdam, the Netherlands

**V. W. V. Jaddoe, MD, PhD, Prof**; Department of Pediatrics, Erasmus University Medical Center Rotterdam, the Netherlands; Department of Epidemiology, Erasmus University Medical Center Rotterdam, the Netherlands; The Generation R Study Group, Erasmus University Medical Center Rotterdam, the Netherlands

**L. Kragt, MSc, PhD**; Department of Oral & Maxillofacial Surgery, Special Dental Care and Orthodontics, Erasmus University Medical Center, Rotterdam, the Netherlands; The Generation R Study Group, Erasmus University Medical Center, Rotterdam, the Netherlands

**Correspondence to**: **A.W. van Meijeren - van Lunteren**, P.O Box 2040, 3000 CA Rotterdam, the Netherlands, phone number: 010-7037733, e-mail: a.vanlunteren@erasmusmc.nl

Children participating in follow up phase at 9 year (n=7,393)

Children with available OHRQoL measure (n=3,796)

Children representing mothers’ ethnic background groups of n >100 (n=3,121)

Dutch n=2,510

Indonesian n=143

Moroccan n=104

Surinamese n=195

Creole n=72

Hindustani n=84

Turkish n=169

**Excluded children (n=675)**

Other ethnic background n=650

Missing n=25

**Excluded children (n=3,597):**

No OHRQoL measure available or more than 3 missing answers

Total cohort (n=9,749)

**Figure 1 – Flowchart showing selection procedure of study population**

**Table 1 - The 11 items of the questionnaire COHIP-ortho**

| During the past 3 month, how often has your child: |
| --- |
| 1. had pain in his/her teeth/toothache *(D1-Oral Health)* |
| 1. had crooked teeth or spaces between his/her teeth *(D1-Oral Health)* |
| 1. had discolored teeth or spots on his/her teeth *(D1-Oral Health)* |
| 1. had bad breath (*D1-Oral Health)* |
| 1. had bleeding gums *(D1-Oral Health)* |
| 1. had difficulty eating foods he/she would like to because of his/her teeth, mouth, or face *(D2-Functional Well-Being)* |
| 1. felt worried or anxious because of his/her teeth mouth or face *(D3-Socio-emotional Well-Being)* |
| 1. not wanted to speak/ read out loud in class because of his/her teeth, mouth, or face *(D4-School/Environment)* |
| 1. been teased, bullied or called names by other children because of his/ her teeth, mouth, or face *(D3-Socio-emotional Well-Being)* |
| 1. felt that he/she was attractive (good looking) because of his/ her teeth, mouth, or face (*D5-Self-image*) |
| 1. had difficulty saying certain words because of his/her teeth or mouth *(D2-Functional Well-Being)* |

Table 2 – Evaluation of medically important differences among ethnic background groups in mean score OHRQoL using Cohen’s effect sizes

|  | Mean OHRQoL (SD)^a^ | Cohen’s effect sizes^b^ |
| --- | --- | --- |
| **Dutch** | 49.17 (3.01) | Ref |
| **Indonesian** | 49.10 (2.87) | 0.02 |
| **Moroccan** | 48.68 (3.59) | 0.15 |
| **Surinamese** | 48.45 (3.66) | 0.22 * |
| **Surinamese-Creole** | 49.14 (3.17) | 0.01 |
| **Surinamese-Hindustani** | 47.86 (4.13) | 0.37 * |
| **Turkish** | 48.17 (4.11) | 0.28 * |
| ^a mean OHRQoL is presented as group mean with corresponding SD. b Cohen’s effect sizes (d) calculated by the difference in mean OHRQoL and divided by a pooled SD. * p ≤ 0.05..p-values are based on pairwise comparisons between group means, using Bonferroni tests.^ | | |

Table 3 – Weighted linear regression models showing the association between mediators (M) and OHRQoL (Y)

|  | Univariate model |
| --- | --- |
| **Caries experience** |  |
| No caries (n=2,385) | Ref |
| Mild caries (n=498) | -0.32 (-0.66 - 0.03) |
| Severe caries (n=238) | **-1.04 (-1.64 - -0.43)***** |
| **Maternal education level** |  |
| Low (n=396) | **-0.54 (-0.95 - -0.13)**** |
| Middle (n=896) | -0.26 (-0.52 - 0.05) |
| High (n=1829) | Ref |
| **Family income** |  |
| Low (n=340) | **-0.76 (-1.21 - -0.32)***** |
| Middle (n=1,113) | -0.24 (-0.48 – 0.01) |
| High (n=3121) | Ref |
| ^Data are presented as weighted least squares regression coefficients (β) with 95% confidence interval (95% CI)., adjusted for age and gender of the child.^  ^**<0.01^  ^***<0.001^ | |

Table 4 – Ordinal logistic regression models showing the associations between ethnic background and every item of the OHRQoL questionnaire (COHIP-ortho)

|  | Model 1 | Model 2 | Model 3 |  |
| --- | --- | --- | --- | --- |
|  |  |  |  |  |
| 1. had pain in his/her teeth/toothache (D1-Oral Health) | | | | |
| Dutch (n=2,510) | Ref | Ref | Ref | |
| Indonesian (n=143) | 0.84 (0.50-1.40) | 0.82 (0.49-1.37) | 0.83 (0.50-1.40) | |
| Moroccan (n=104) | **0.47 (0.29-0.77)**** | 0.64 (0.38-1.08) | 0.72 (0.42-1.23) | |
| Surinamese (n=195) | **0.52 (0.36-0.76)***** | **0.57 (0.39-0.84)**** | **0.61 (0.41-0.91)*** | |
| Turkish (n=169) | **0.42 (0.29-0.62)**** | **0.54 (0.36-0.80)**** | **0.58 (0.38-0.89)*** | |
| 1. had crooked teeth or spaces between his/her teeth (D1-Oral Health) | | | | |
| Dutch (n=2,510) | Ref | Ref | Ref | |
| Indonesian (n=143) | 1.03 (0.73-1.46) | 1.04 (0.73-1.46) | 1.04 (0.74-1.47) | |
| Moroccan (n=104) | 0.97 (0.65-1.44) | 0.92 (0.61-1.39) | 0.92 (0.60-1.41) | |
| Surinamese (n=195) | 0.92 (0.69-1.23) | 0.91 (0.68-1.22) | 0.90 (0.67-1.23) | |
| Turkish (n=169) | 1.07 (0.77-1.48) | 1.03 (0.73-1.45) | 1.02 (0.72-1.45) | |
| 1. had discolored teeth or spots on his/her teeth (D1-Oral Health) | | | | |
| Dutch (n=2,510) | Ref | Ref | Ref | |
| Indonesian (n=143) | 0.90 (0.61-1.33) | 0.90 (0.61-1.33) | 0.91 (0.62-1.33) | |
| Moroccan (n=104) | 0.80 (0.53-1.23) | 0.85 (0.55-1.33) | 0.91 (0.57-1.44) | |
| Surinamese (n=195) | **0.63 (0.47-0.86)**** | **0.64 (0.47-0.87)**** | **0.67 (0.48-0.92)*** | |
| Turkish (n=169) | 0.84 (0.59-1.20) | 0.89 (0.61-1.33) | 0.93 (0.63-1.36) | |
| 1. had bad breath (D1-Oral Health) | | | | |
| Dutch (n=2,510) | Ref | Ref | Ref | |
| Indonesian (n=143) | 1.26 (0.85-1.88) | 1.26 (0.85-1.87) | 1.25 (0.84-1.86) | |
| Moroccan (n=104) | 1.18 (0.76-1.85) | 1.30 (0.82-2.05) | 1.55 (0.97-2.49) | |
| Surinamese (n=195) | **0.53 (0.40-0.70)***** | **0.54 (0.40-0.72)***** | **0.62 (0.46-0.84)**** | |
| Turkish (n=169) | **0.67 (0.49-0.93)*** | 0.73 (0.52-1.02) | 0.88 (0.62-1.24) | |
| 1. had bleeding gums (D1-Oral Health) | | | | |
| Dutch (n=2,510) | Ref | Ref | Ref | |
| Indonesian (n=143) | 0.89 (0.60-1.32) | 0.89 (0.60-1.32) | 0.91 (0.61-1.35) | |
| Moroccan (n=104) | **0.57 (0.38-0.86)**** | 0.66 (0.43-1.01) | 0.78 (0.50-1.21) | |
| Surinamese (n=195) | **0.49 (0.36-0.66)***** | **0.50 (0.37-0.68)***** | **0.56 (0.41-0.76)***** | |
| Turkish (n=169) | **0.49 (0.35-0.67)***** | **0.56 (0.40-0.78)**** | **0.64 (0.45-0.92)*** | |
| 1. had difficulty eating foods he/she would like to because of his/her teeth, mouth, or face (D2-Functional Well-Being) | | | | |
| Dutch (n=2,510) | Ref | Ref | Ref | |
| Indonesian (n=143) | 0.73 (0.42-1.24) | 0.72 (0.42-1.24) | 0.73 (0.43-1.26) | |
| Moroccan (n=104) | 0.64 (0.35-1.17) | 0.83 (0.44-1.53) | 0.94 (0.49-1.78) | |
| Surinamese (n=195) | **0.36 (0.25-0.53)***** | **0.38 (0.26-0.56)***** | **0.43 (0.29-0.63)***** | |
| Turkish (n=169) | **0.44 (0.29-0.68)***** | **0.54 (0.35-0.85)**** | **0.61 (0.38-0.98)*** | |
| 1. felt worried or anxious because of his/her teeth mouth or face (D3-Socio-emotional Well-Being) | | | | |
| Dutch (n=2,510) | Ref | Ref | Ref | |
| Indonesian (n=143) | 0.77 (0.53-1.13) | 0.76 (0.52-1.12) | 0.78 (0.53-1.15) | |
| Moroccan (n=104) | 0.84 (0.54-1.32) | 1.00 (0.63-1.60) | 1.11 (0.69-1.80) | |
| Surinamese (n=195) | 0.75 (0.54-1.05) | 0.78 (0.56-1.09) | 0.84 (0.59-1.18) | |
| Turkish (n=169) | 0.89 (0.62-1.28) | 1.03 (0.70-1.51) | 1.13 (0.76-1.69) | |
| 1. not wanted to speak/ read out loud in class because of his/her teeth, mouth, or face (D4-School/Environment) | | | | |
| Dutch (n=2,510) | Ref | Ref | Ref | |
| Indonesian (n=143) | 0.64 (0.19-2.10) | 0.63 (0.19-2.08) | 0.63 (0.19-2.09) | |
| Moroccan (n=104) | **0.14 (0.07-0.28)***** | **0.16 (0.07-0.36)***** | **0.23 (0.10-0.54)**** | |
| Surinamese (n=195) | 0.53 (0.20-1.37) | 0.56 (0.21-1.44) | 0.75 (0.28-2.01) | |
| Turkish (n=169) | **0.20 (0.10-0.40)***** | **0.23 (0.11-0.50)***** | **0.33 (0.15-0.76)**** | |
| 1. been teased, bullied or called names by other children because of his/ her teeth, mouth, or face (D3-Socio-emotional Well-Being) | | | | |
| Dutch (n=2,510) | Ref | Ref | Ref | |
| Indonesian (n=143) | 1.58 (0.49-5.06) | 1.57 (0.49-5.02) | 1.56 (0.49-5.01) | |
| Moroccan (n=104) | **0.22 (0.12-0.40)***** | **0.29 (0.15-0.55)***** | **0.36 (0.18-0.71)**** | |
| Surinamese (n=195) | **0.45 (0.25-0.80)**** | **0.47 (0.26-0.86)*** | **0.55 (0.30-0.97)*** | |
| Turkish (n=169) | 0.54 (0.27-1.06) | 0.68 (0.33-1.40) | 0.86 (0.40-1.81) | |
| 1. felt that he/she was attractive (good looking) because of his/ her teeth, mouth, or face (D5-Self-image) | | | | |
| Dutch (n=2,510) | Ref | Ref | Ref | |
| Indonesian (n=143) | 1.06 (0.75-1.50) | 1.06 (0.75-1.49) | 1.08 (0.76-1.52) | |
| Moroccan (n=104) | **0.66 (0.45-0.95)*** | 0.66 (0.45-1.02) | 0.81 (0.54-1.21) | |
| Surinamese (n=195) | **0.55 (0.41-0.74)***** | **0.55 (0.42-0.74)***** | **0.66 (0.49-0.88)**** | |
| Turkish (n=169) | 0.97 (0.71-1.32) | 0.98 (0.71-1.35) | 1.20 (0.86-1.68) | |
| 1. had difficulty saying certain words because of his/her teeth or mouth (D2-Functional Well-Being) | | | | |
| Dutch (n=2,510) | Ref | Ref | Ref | |
| Indonesian (n=143) | 5.31 (0.73-38.40) | 5.31 (0.83-33.92) | 5.38 (0.51-14.76) | |
| Moroccan (n=104) | **0.27 (0.15-0.52)***** | **0.31 (0.16-0.62)**** | **0.35 (0.17-0.71)**** | |
| Surinamese (n=195) | 0.75 (0.37-1.52) | 0.77 (0.14-1.57) | 0.81 (0.39-1.68) | |
| Turkish (n=169) | **0.36 (0.20-0.63)***** | **0.41 (0.22-0.75)**** | **0.44 (0.23-0.85)*** | |
| ^Data are presented as Odds Ratios (OR) with 95% confidence interval (95% CI).  Model 1 is adjusted for age and gender of the child, Model 2 is additionally adjusted for caries experience, Model 3 is additionally adjusted for caries experience, family income and educational level of the mother.^  ^*<0.05^  ^**<0.01^  ^***<0.001^ | | | | |

Table 5 - Non-response analysis comparing characteristics of the participating population with the non-participating population

|  | Included (N=3,121) | Excluded (N=4,272) | *p-*value |
| --- | --- | --- | --- |
| Individual characteristics |  |  |  |
| Child’s gender |  |  | 0.711 |
| Boys, *n* (%) | 1,573 (50.4) | 2,134 (50.0) |  |
| Girls, *n* (%) | 1,548 (49.6) | 2,137 (50.0) |  |
| *Missings, n (%)* | *0 (0.0)* | *1 (0.0)* |  |
| Child’s age |  |  | < 0.001 |
| Mean (SD) | 9.85 (0.36) | 9.93 (0.41) |  |
| *Missings, n (%)* | *0 (0.0)* | *3,252 (76.1)* |  |
| Child’s caries experience, *n* (%) |  |  | < 0.001 |
| No caries (dmft 0) | 1,778 (76.4) | 1,677 (64.1) |  |
| Mild caries (dmft 1-3) | 368 (15.8) | 548 (12.8) |  |
| Severe caries (dmft >3) | 181 (7.8) | 393 (15.0) |  |
| *Missings, n (%)* | *794 (25.4)* | *1,654 (61.3)* |  |
| Child’s OHRQoL |  |  | < 0.001 |
| Mean (SD) | 49.05 (3.15) | 48.31 (3.52) |  |
| *Missings, n (%)* | *0 (0.0)* | *3,597 (84.2)* |  |
| Family characteristics |  |  |  |
| Maternal education level, *n* (%) |  |  | < 0.001 |
| Low | 366 (12.1) | 1,112 (30.3) |  |
| Middle | 852 (28.3) | 1,184 (32.0) |  |
| High | 1,795 (59.6) | 1,396 (37.7) |  |
| *Missings, n (%)* | *108 (3.5)* | *570 (13.3)* |  |
| Household income, *n* (%) |  |  | < 0.001 |
| Low (< €1,600) | 308 (10.6) | 420 (24.3) |  |
| Middle (€1,600-3,600) | 1,032 (35.4) | 657 (38.1) |  |
| High (> €3,600) | 1,579 (50.6) | 649 (37.6) |  |
| *Missings, n (%)* | *202 (6.5)* | *2,546 (59.6)* |  |
| ^Numbers are presented as absolute numbers for categorical variables or as mean (SD) for continuous variables. P-values are estimated based on chi-square tests and independent samples T-tests.^ | | | |

**Table 6 – Differences in OHRQoL of children between groups were mothers have the same versus different ethnic backgrounds than fathers or children**

| Child’s OHRQoL Mean (SD) |  |
| --- | --- |
| Mother and children with same ethnic background (n=2,690) | 49.05 (3.17) |
| Mother and children with not the same ethnic background (n=431) | 49.05 (3.04) |
| Mother and father with the same ethnic background (n=2,414) | 49.04 (3.16) |
| Mothers and fathers with not the same ethnic background (n=635) | 49.20 (2.93) |
| ^Numbers are presented as absolute numbers for categorical variables or as mean (SD).^ | |
